# Successful Thoracic Duct Embolization Following Fontan-Related Chylothorax in a Six-Year-Old Girl: A Case Report

**DOI:** 10.7759/cureus.63005

**Published:** 2024-06-23

**Authors:** Shailesh Wandile, Jayant D Vagha, Ajinkya Wazurkar, Sham Lohiya, Dinesh V Hinge, Chaitanya Kumar Javvaji, Prashant B Khartade, Pooja B Nagrale, Anirudh Kommareddy, Prachita Agrawal

**Affiliations:** 1 Pediatrics, Jawaharlal Nehru Medical College, Datta Meghe Institute of Higher Education and Research, Wardha, IND; 2 Interventional Radiology, Jawaharlal Nehru Medical College, Datta Meghe Institute of Higher Education and Research, Wardha, IND

**Keywords:** pleural effusion, thoracic duct embolisation, chylothorax, fontan operation, ventricular septal defect (vsd)

## Abstract

Chylothorax is a severe complication following the Fontan procedure, causing significant morbidity and mortality due to nutritional depletion and fluid loss. We present a case involving a six-year-old girl with tricuspid atresia, atrial septal defect (ASD), ventricular septal defect (VSD), and severe pulmonary stenosis (PS), presenting with fever, non-productive cough, and increased work of breathing. Cyanosis was noted, improving with oxygen. Imaging revealed bilateral pleural effusion, with pleural fluid analysis confirming chylothorax. Despite normal laboratory reports, retrograde transvenous lymphangiography indicated thoracic duct leakage. The patient underwent successful thoracic duct embolization, resulting in the resolution of the effusion and stabilization of her condition. She was discharged in a stable state, with follow-up care.

## Introduction

Chylothorax after congenital heart disease (CHD) surgery may be caused by damage to the thoracic duct, lymphatic discontinuity, and increased systemic venous pressures greater than those in the thoracic duct [1-3]. Patients with heart disease who experience post-operative chylothorax face a serious risk of severe morbidity and mortality [4-11]. Patients with chylothorax often require prolonged ventilator support and extended hospital stays, and they are at high risk for hospital-acquired infections, severe weight loss, and mortality. Symptoms arise when large volumes of chylous fluid accumulate, leading to nutritional depletion and significant fluid and electrolyte losses over time [12,13]. This case report details the clinical course and successful treatment of a six-year-old girl who developed chylothorax following the Fontan procedure. The purpose of this case report is to highlight the serious risks and management challenges associated with post-operative chylothorax in pediatric patients with CHD undergoing complex surgeries like the Fontan procedure. It emphasizes the importance of early identification and diagnosis of chylothorax, the clinical manifestations to watch for, and the effective intervention of thoracic duct embolization with n-butyl cyanoacrylate glue. By documenting a successful case, this report aims to provide insights into improved management strategies, ultimately enhancing patient outcomes and reducing morbidity and mortality in similar clinical scenarios.

## Case presentation

A six-year-old girl presented with a seven-day history of cough and increased work of breathing, accompanied by a two-day history of fever. The cough was non-productive, predominantly occurring during the day, and was associated with post-tussive vomiting. The fever was insidious in onset, intermittent, moderate grade, and relieved by medication. The patient had a recurrent history of respiratory tract infections and was previously diagnosed with tricuspid atresia, atrial septal defect (ASD), severe pulmonary stenosis (PS), and a ventricular septal defect (VSD). She had undergone the Fontan procedure two months prior.

**Figure 1:**
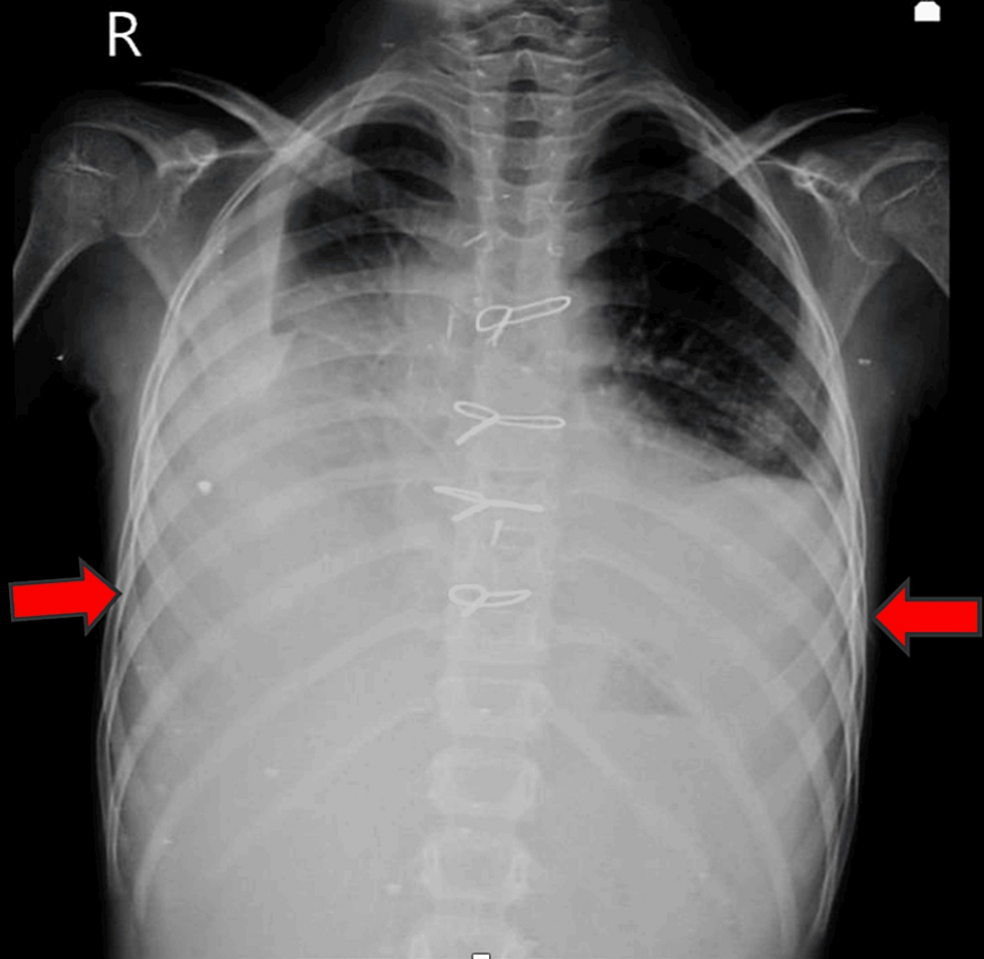
Chest X-ray showing bilateral pleural effusion (red arrows)

On examination, the patient had a heart rate of 94 beats per minute, a respiratory rate of 44 breaths per minute, and an oxygen saturation of 84% in all four limbs, with noted cyanosis. Respiratory examination revealed tachypnea, chest retractions, and reduced air entry bilaterally. Cardiovascular examination identified a pan-systolic murmur, while other systems were unremarkable. A chest X-ray indicated bilateral pleural effusion (Figure 1).

**Figure 2:**
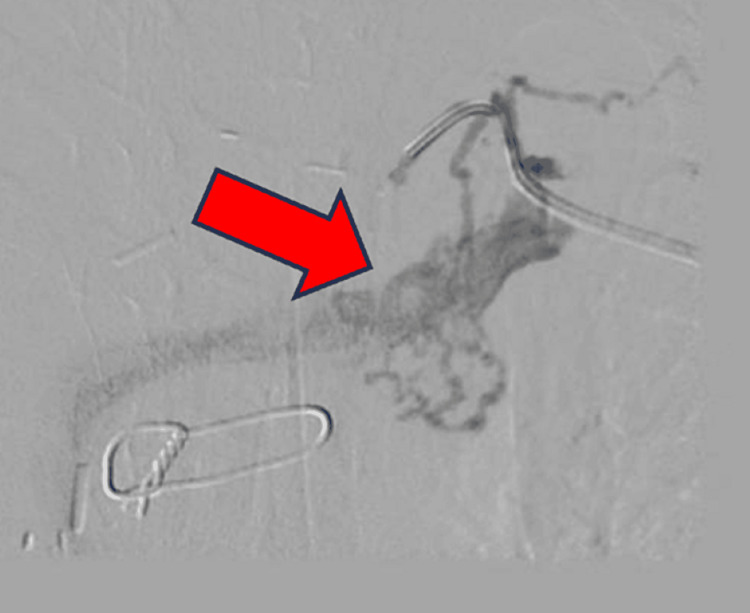
Retrograde transvenous lymphangiography showing thoracic duct leak (red arrow)

Due to escalating respiratory distress, pleural fluid tapping was performed on the right side. The analysis revealed lactic dehydrogenase at 239 IU/L, protein at 4.7 g/dL, the potential of hydrogen (pH) at 7.5, glucose at 92 mg/dL, and triglycerides at 251 mg/dL, suggestive of chylothorax. Pleural fluid culture revealed no growth. Trunaat of pleural fluid was negative. The Mantoux test was negative, and other laboratory investigations were within normal limits. A 2D echocardiogram showed good flow in the Fontan and fenestration, along with tricuspid atresia, PS, VSD, ASD, and bilateral pleural effusion. Repeated pleural tapping on the right side was necessary due to persistent respiratory distress. Retrograde transvenous lymphangiography revealed leakage in the upper third of the thoracic duct on the right side (Figure 2).

Under all aseptic precautions, access to the left cephalic vein was achieved using a 4F Heath catheter. A venogram was then performed with a Cobra catheter. Selective cannulation of the thoracic duct was successfully carried out. Following this, a Progreat microcatheter (Terumo, Somerset, NJ, USA) was placed, demonstrating contrast reflux from the thoracic duct. Embolization was performed using a mixture of 25% n-butyl cyanoacrylate glue and Lipiodol (Video 1). The complete angiogram post-embolization demonstrated no reflux from the thoracic duct. The procedure was uneventful.

**Video 1:** Transvenous retrograde thoracic duct embolization showing n-butyl cyanoacrylate glue in the thoracic duct

**Figure 3:**
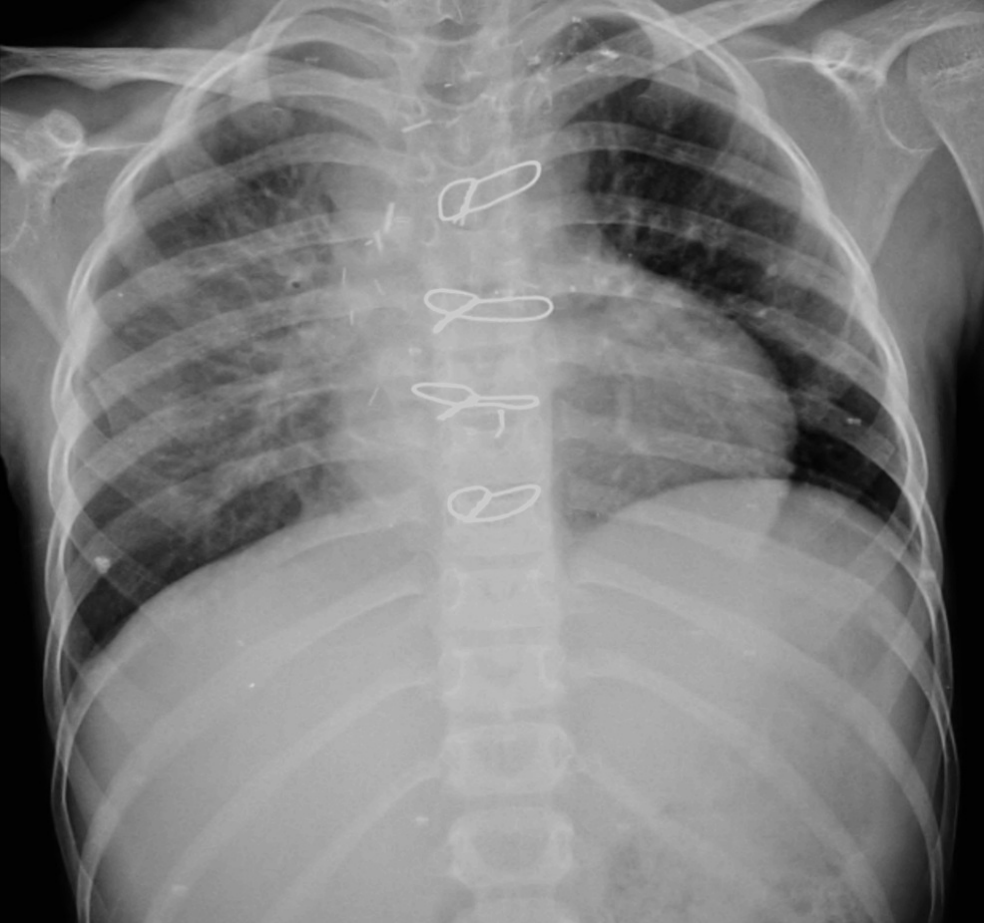
Chest X-ray post-embolization showing resolved bilateral pleural effusion

Following the procedure, oxygen support was gradually reduced as the patient’s condition improved. A repeat chest X-ray showed no pleural effusion and good bilateral air entry (Figure 3). The patient was vitally and hemodynamically stable and hence was discharged.

## Discussion

Chylous pleural effusion stands out as a significant and worsening complication following surgery, particularly in the post-operative period [14,15]. Although relatively rare after CHD repairs, chylothorax can lead to substantial morbidity [16]. Increased intrathoracic lymphatic pressure or surgical discontinuity of the thoracic duct or its branches may cause post-operative lymphatic fluid leakage into the pleural space.

A delayed diagnosis is associated with prolonged drainage duration, significant loss of lipids and proteins, immunosuppression, prolonged dependence on chest tubes and intravenous access, and extended hospital stays. Early application of treatment modalities is essential, as delayed initiation of octreotide therapy in patients with higher chyle drainage has shown limited improvement. It is clear that increased knowledge of the possibility of post-operative chylothorax is essential for early detection of this potentially dangerous complication and quick patient care [2].

Since the thoracic duct has thin walls and is colorless, it is challenging to locate and may accidentally be injured during investigative or surgical procedures in the posterior mediastinum. Chylothorax is the outcome of lymph leaking into the thoracic cavity and pleural space when the thoracic duct is punctured, either by trauma or thoracic surgery. Various intrathoracic operations can cause chylothorax; however, some congenital defects are more likely to result in this complication [4,9].

Procedures that elevate systemic venous pressure, particularly Fontan-type procedures and right ventricular dysfunction following tetralogy of Fallot repair, carry an increased risk of post-operative chylothorax [2,4]. The occurrence of Fontan surgery and subsequent chylothorax in this case further corroborates findings from previous studies.

It is widely known that there has been a recent rise in the incidence of post-operative chylothorax, from the previously reported 1% or less to 2.5% to 4.7% [12]. After surgery for CHD, thoracic duct damage, accessory lymphatic disruption, and elevated systemic vein pressure above that of the thoracic duct have all been suggested as potential causes of chylothorax [2]. Post-operative chylothorax is a danger associated with any procedures that raise the risk of elevated systemic venous pressure. Post-operative chylothorax has been specifically linked to bidirectional cavopulmonary shunt operation, Fontan-type surgeries, and right ventricular failure following tetralogy of Fallot repair. The earlier research was further supported by a study by Akbari et al. that showed a substantial correlation between Fontan surgery and post-operative chylothorax. Chylous drainage is impaired, and chyle loss is increased during cavopulmonary anastomosis in the Fontan procedure due to both mechanical injury to the thoracic duct during surgical manipulation and systemic venous hypertension with subsequent back-up of pressure into the thoracic duct [17]. In our case, the chylothorax was due to mechanical injury to the thoracic duct.

Conservative treatment is typically the first-line approach, especially effective if the drain output is less than 1000 mL/day. The duration of this treatment varies, generally lasting 7-14 days in adults and can be longer in children, though a shorter attempt (up to three days) is advised in cases of high-volume leakage due to the delicate fluid and electrolyte balance. Dietary adjustments, such as incorporating medium-chain triglycerides, are often insufficient on their own. Complete parenteral nutrition is usually the initial therapeutic step, particularly post-operatively. Somatostatin or octreotide may be used as adjuncts, especially in children, though the evidence of their effectiveness is inconsistent. Thoracocentesis or drainage is utilized to aid lung expansion and improve pulmonary function, while irradiation or chemotherapy might be necessary as part of treating the underlying disease [18].

When conservative treatment fails or is unlikely to succeed, surgical options are considered. Thoracic duct ligation, with a success rate of about 95%, is the most common surgical treatment. If the thoracic duct is unidentifiable, mass ligation may be performed. Suturing the thoracic duct leak is technically challenging and not favored over ligation. Suturing pleural defects, pleurodesis, and pleurectomy are other surgical strategies, the latter two being particularly relevant in cases of malignancy or when the lung cannot expand. Chylovenous anastomosis is rarely performed due to its poor success rate, while a pleuroperitoneal shunt is an option in refractory cases but is contraindicated if there are chylous ascites. An external catheter or intermittent drainage may be considered if pleurodesis is not feasible [19,20].

Interventional radiology offers additional treatment avenues, particularly for hepatic chylothorax associated with cirrhosis or portal hypertension through a transjugular intrahepatic portosystemic shunt (TIPS). Lymphography, sometimes leading to spontaneous occlusion of a chyle fistula, and percutaneous closure of the thoracic duct through catheterization and embolization, are other options. These procedures are technically demanding and not universally available, but they are usually successful if the thoracic duct can be intubated [19].

## Conclusions

In conclusion, early recognition and management of post-operative chylothorax, a rare yet significant complication following CHD repairs, are critical. Delayed diagnosis can lead to prolonged drainage, substantial nutrient losses, and increased hospital stays. Prompt initiation of treatment is essential for optimal outcomes; understanding risk factors, such as specific surgical procedures and congenital malformations, aids in timely intervention. Vigilant monitoring and swift action can mitigate complications and improve patient outcomes.
